# Application of a Novel Adaptive Med Fault Diagnosis Method in Gearboxes

**DOI:** 10.3390/e21111106

**Published:** 2019-11-12

**Authors:** Wenhua Du, Xiaoming Guo, Xiaofeng Han, Junyuan Wang, Jie Zhou, Zhijian Wang, Xingyan Yao, Yanjun Shao, Guanjun Wang

**Affiliations:** 1 College of mechanical engineering, North University of China, Taiyuan 030051, China; dwh@nuc.edu.cn (W.D.); s1802011@st.nuc.edu.cn (X.G.); s1702044@st.nuc.edu.cn (X.H.); wjy@nuc.edu.cn (J.W.); s1702015@st.nuc.edu.cn (J.Z.); syj@nuc.edu.cn (Y.S.); 2 School of Computer Science and Information Engineering, Chongqing Technology and Business University, Chongqing 400067, China; xyyao@ctbu.edu.cn; 3 Collage of Information Science & Technology, Hainan University, Haikou 570228, China

**Keywords:** fault diagnosis, minimum entropy deconvolution, firefly optimization algorithm, singular spectrum decomposition

## Abstract

Minimum entropy deconvolution (MED) is not effective in extracting fault features in strong noise environments, which can easily lead to misdiagnosis. Moreover, the noise reduction effect of MED is affected by the size of the filter. In the face of different vibration signals, the size of the filter is not adaptive. In order to improve the efficiency of MED fault feature extraction, this paper proposes a firefly optimization algorithm (FA) to improve the MED fault diagnosis method. Firstly, the original vibration signal is stratified by white noise-assisted singular spectral decomposition (SSD), and the stratified signal components are divided into residual signal components and noisy signal components by a detrended fluctuation analysis (DFA) algorithm. Then, the noisy components are preprocessed by an autoregressive (AR) model. Secondly, the envelope spectral entropy is proposed as the fitness function of the FA algorithm, and the filter size of MED is optimized by the FA algorithm. Finally, the preprocessed signal is denoised and the pulse enhanced with the proposed adaptive MED. The new method is validated by simulation experiments and practical engineering cases. The application results show that this method improves the shortcomings of MED and can extract fault features more effectively than the traditional MED method.

## 1. Introduction

Fault diagnosis is a hot topic in recent years, and many scholars have studied it [1,2,3,4,5]. Fault diagnosis of rotating machinery is the focus of this research. When the rotating machinery fails, the fault signal collected by sensors usually presents non-linear and non-stationary characteristics [6,7,8,9,10]. The transmission process of rolling bearing fault source signals can be regarded as a linear convolution mixing process between the source signal and channel, and the extraction of the fault’s original shock signal can be regarded as a deconvolution process [11,12,13,14,15,16,17]. From this point-of-view, in 1978, R.A. Wiggins [18] first proposed minimum entropy deconvolution (MED) in the field of blind convolution and successfully applied it to seismic wave processing. Subsequently, many experts and scholars have carried out research on this issue. Donald [19] further improved the MED method and gave him a general explanation. MED is a deconvolution filter, which maximizes the kurtosis by searching for the inverse filter to offset the influence of transmission path. It can not only enhance the impact component, but also reduce the noise of the signal. Edno [20] first used this method to enhance the signal impact caused by spalling and crack failure in gearboxes. Sawalhi [21] and others applied it to fault diagnosis of rolling bearings. Li et al. [22] proposed a method of combining monostable stochastic resonance with minimum entropy deconvolution based on time-delay feedback for fault diagnosis of rolling bearings. Liu et al. [23] used the blind deconvolution method to filter the noise components of infrared spectroscopy and calculated the entropy value to determine the effect of noise reduction. Quantitative and qualitative analysis showed that the method was superior to traditional noise reduction methods. Sawalhi and Randall have studied the MED algorithm deeply. They use MED to reduce the noise of vibration signals, and then calculate the fast kurtosis. By diagnosing and analyzing the faults of the inner and outer rings of the bearings, the diagnostic ability of spectral kurtosis is improved, and the effectiveness of this method is verified. MED can effectively reduce noise and enhance impact components. However, the effect of enhancing impact and noise reduction is poor in strong noise environments and vulnerable to the influence of filter size. Therefore, it is necessary to improve the anti-noise ability of MED and optimize its filter size.

Empirical mode decomposition (EMD) is a signal time–frequency analysis method proposed by N. E. Huang in 1998 [24]. EMD has the characteristics of orthogonality, completeness and self-adaptability, and has been widely used in signal processing and fault diagnosis. However, the existence of modal aliasing and endpoint effect limits its further promotion [25,26,27,28,29,30,31,32]. Ensemble empirical mode decomposition (EEMD) proposed by Wu and Huang in 2009 [33] can adaptively decompose complex mixed signals into a series of intrinsic mode functions (IMFs), which distribute different frequencies on different IMFs to achieve noise reduction. Adding white noise can also partially reduce modal aliasing. However, the results of EEMD decomposition contain redundant noise components, which require a large number of set experiments to eliminate redundant noise components. This process is time-consuming. The phenomenon of mode aliasing still exists in EEMD, especially in the strong noise environment, and its signal processing effect is very unsatisfactory [34,35,36,37,38,39]. Singular spectral analysis (SSA) is a mathematical analysis method based on principal component analysis for non-parametric spectral estimation. The main process of traditional singular spectrum analysis is that the original time series is decomposed into several parts, and then a new time series is reconstructed according to certain criteria. However, this method has the problem of reducing the energy of the residual sequence in the iteration. In 2014, Pietro [40] proposed a new method of fault signal decomposition on the basis of SSA, singular spectrum decomposition (SSD). This method improves the construction method of the trajectory matrix and the reconstruction method of the component sequence, makes up for the shortcomings of energy reduction in the iteration of residual sequences and realizes the adaptive reconstruction process of the signal, which provides a new idea for processing non-stationary and non-linear signals. Similar to EMD, SSD decomposition is based on extracting signal components related to various inherent time scales. Compared with EMD, SSD can relieve the modal aliasing and provide accurate separation between the intermittent components at the transition point. However, there are still some problems in SSD decomposition, such as modal aliasing and a large number of unrecognizable pseudo-components at high-frequency components. The fault information of SSD decomposition is easily submerged under strong noise, which makes it difficult to extract feature frequencies.

In order to remedy the shortcomings of MED, a new adaptive fault diagnosis method for MED is proposed in this paper. Firstly, Gauss white noise is added to the original signal several times, and then the signal with white noise is decomposed by SSD to get multiple signal components. Based on the principle that the statistical mean value of uncorrelated random sequence is 0, the signal components corresponding to the above steps are averaged to eliminate the influence of multiple additions of white Gaussian noise on the signal components, and the final decomposition results are obtained. A detrended fluctuation analysis (DFA) algorithm is used to calculate the scaling exponents of each signal component, and to judge whether it is a noisy component or a residual component. Then the noisy signal component is processed by an autoregressive (AR) model to reduce the stationary part of the signal, which can be predicted linearly and can separate the impulse component of the vibration signal. At the same time, envelope spectral entropy is used as the fitness function of the firefly optimization algorithm, and the firefly optimization algorithm is used to optimize the size of the MED filter, so that the filter size of the MED algorithm can be adaptively selected, and the noise reduction and pulse enhancement effect of MED are further improved. Finally, the noisy signal components processed by adaptive MED, as well as the de-noised signal components and residual components, are reconstructed to get the final results.

This article is arranged as follows. In Section 2, the basic principles of MED, SSD, DFA, AR and the new methods proposed in this paper are briefly introduced. Section 3 compares several traditional methods and new methods through simulation experiments, and analyzes the results. In Section 4, a new method is used to deal with the fault of gearbox in practical engineering cases, and the results are analyzed. Finally, Section 5 is the summary part, which summarizes the whole research and puts forward the prospects for the future.

## 2. Principle of New Methods and Related Theories 

### 2.1. Principle of MED

Equipment failure can cause a shock signal, but the original "certainty" of the shock signal is destroyed with the influence of transmission path, which leads to the increase of signal entropy. In order to restore the original shock state of the signal, it is necessary to estimate the inverse transfer function and reduce the entropy value. Assume that the fault signal expression is as follows:

(1) y(n)=h(n)∗x(n)+e(n).

Assuming that the input x(n) is the impulse sequence of the fault signal and h(n) is the impulse response function of the transmission path, the influence of the noise signal e(n) on the system is neglected for the time being. The deconvolution process is to find an inverse filter w(n) of order L, which can restore the lagged output y(n) to the input x(n) through the inverse filter. The expression of the deconvolution process is as follows:

(2) x(n)=w(n)∗y(n).

Wiggins evaluates the entropy value by the sequence norm obtained after deconvolution in order to solve the optimal result. The expression is as follows:

(3) $$O_{2}^{4}(w(n))=\frac{\sum_{i=1}^{N}x^{4}(i)}{{\left[\sum_{i=1}^{N}x^{2}(i)\right]}^{2}}.$$

The purpose of the MED algorithm is to find the optimal inverse filter w(n) to minimize the entropy after filtering; that is, to maximize the norm $O_{2}^{4}(w(n))$, and to ensure that the first derivative of the above equation is zero:

(4) $$\frac{\partial O_{2}^{4}(w(n))}{\partial w(n)}=0.$$

According to Equation (2),

(5) $$x(n)=w(n)*y(n)=\sum_{l=1}^{L}w(n)y(n-l),$$

where L is the size of the inverse filter w(n). We can get the derivative on both sides of Equation (5):

(6) $$\frac{\partial w(n)}{\partial w(l)}=y(n-l).$$

According to Equation (6) and further calculation of Equation (3), it can be obtained that

(7) $$\left[\sum_{n=1}^{N}x^{2}(n)/\sum_{n=1}^{N}x^{4}(n)\right]\sum_{n=1}^{N}x^{3}(n)y(n-l)=\sum_{p=1}^{L}w(p)\sum_{n=1}^{N}y(n-l)y(n-p).$$

Let $b=\left[\sum_{n=1}^{N}x^{2}(n)/\sum_{n=1}^{N}x^{4}(n)\right]\sum_{n=1}^{N}x^{3}(n)y(n-l)$, $w=\sum_{p=1}^{L}w(p)$, $A=\sum_{n=1}^{N}y(n-l)y(n-p)$, then Equation (7) can be written as the following matrix expression:

(8) b=Aw

where b is the cross-correlation matrix of the input and output of the inverse filter, A is the Toeplitz autocorrelation matrix of the input of the inverse filter and w is the parameter of the inverse filter. According to Equation (8), the inverse filter matrix W is solved by iteration method

(9) $$W=A^{-1}b.$$

**Figure 1:**
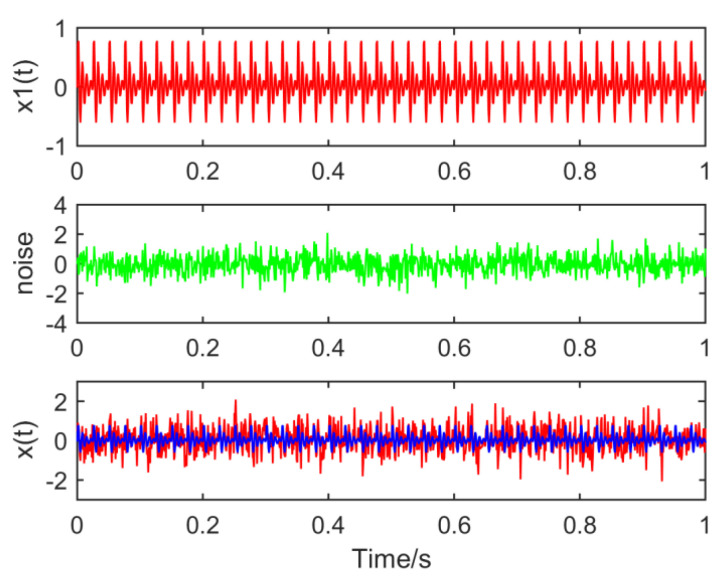
Waveform of the simulated signal.

The following simulation signal is constructed to verify the necessity of optimizing the size of MED filter:

(10) $$x(t)=A_{m}*\exp(-\frac{g}{T_{m}})\sin(2\pi f_{c}t)+noise$$

where $A_{m}=1$, $T_{m}=0.025$, $f_{c}=150Hz$ and the noise amplitude is 0.4, as shown in Figure 1.

**Figure 2:**
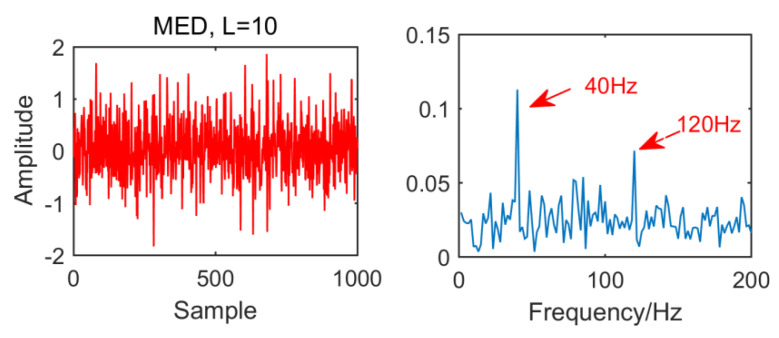
Time domain waveform and envelope spectrum when L = 10.

**Figure 3:**
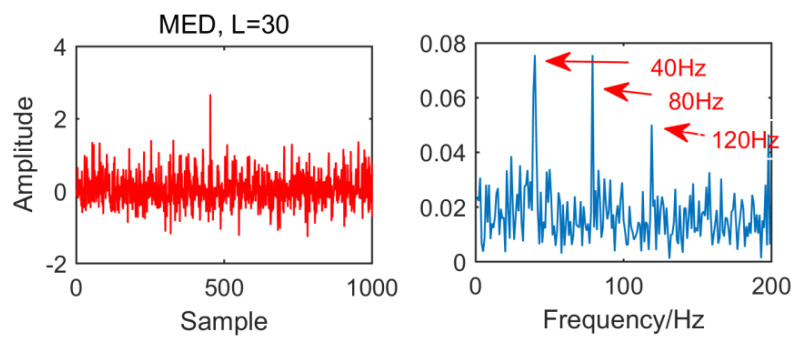
Time domain waveform and envelope spectrum when L = 30.

**Figure 4:**
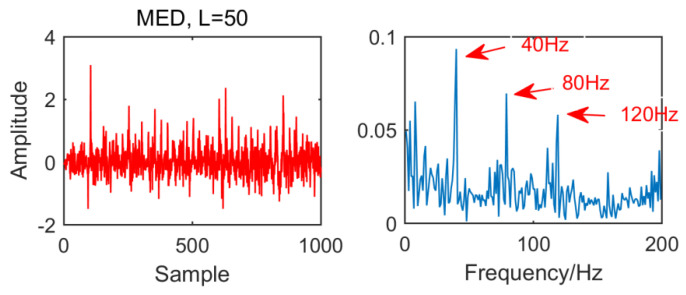
Time domain waveform and envelope spectrum when L = 50.

**Figure 5:**
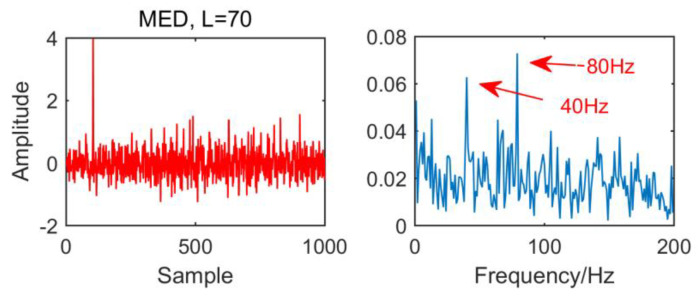
Time domain waveform and envelope spectrum when L = 70.

From the time domain waveform and envelope spectra in Figure 2, Figure 3, Figure 4 and Figure 5, it can be seen that when the size of the MED filter is different, the noise reduction effect of MED on the same fault vibration signal is also different. So, we can conclude that different vibration signals need to have the optimal MED filter size to achieve the best noise reduction effect. In order to improve the noise reduction and pulse enhancement effect of MED for vibration signals, it is necessary to optimize the filter size of MED so that it can choose the appropriate filter size adaptively for different vibration signals.

### 2.2. Principle of SSD

Singular spectral decomposition (SSD) is a new adaptive signal processing method recently proposed. It can decompose the non-linear and non-stationary signals from a high frequency to a low frequency into the sum of several singular spectral components (SSC) and residual terms. The specific process is as follows:

First, a new trajectory matrix is constructed. For a time series x(n), its data length and embedding dimensions are *N* and *M*, respectively. It is constructed as a matrix of *X* of *N* columns and *M* rows, and the *i*-th row of matrix *X* is $X_{i}=(x(i),\cdots x(N),x(1),\cdots x(i-1))$, and i=1,⋯,M; that is, matrix $X={\left[x_{1}^{T},x_{2}^{T},\cdots ,x_{M}^{T}\right]}^{T}$. Selecting K=N−M+1, the lower-right corner of matrix *X* is moved to the upper-left position of matrix *X*, and the improved trajectory matrix X(N×K) is obtained. The improved trajectory matrix can enhance the vibration component of the original signal and make the residual component decrease after iteration.

The embedding dimension *M* is selected adaptively. Considering the defect, SSA chooses the embedding dimension according to experience, and the adaptive rule is used to select the embedding dimension M used in the *j*-th iteration. Firstly, the power spectral density (PSD) of the residual component $v_{j}(n)$ at the *j*-th iteration is calculated, where the residual component $v_{j}(n)$ is

(11) $$v_{j}(n)=x(n)-\sum_{k=1}^{j-1}v_{k}(n)(v_{0}(n)=x(n)).$$

The frequency $f_{\max}$ corresponding to the maximum peak value in the PSD is then estimated. In the first iteration, if the normalized frequency $f_{\max}/F_{s}$ is less than a given threshold of 10^−3^, the residual is considered as a large trend term, and M is set to N/3, where $F_{s}$ is the sampling frequency. Otherwise, when the number of iterations *J* > 1, the embedding dimension is set to $M=1.2F_{s}/f_{\max}$, which improves the analysis effect of SSA.

The *j*-th component signal is reconstructed in the order of high frequency to low frequency. In the first iteration, if a large trend item is detected, only the first or so feature cards are used to obtain $g^{\left(1\right)}(n)$, so that $X_{1}=\sigma_{1}\mu_{1}v_{1}^{T}$ and $g^{\left(1\right)}(n)$ can be obtained from the diagonal average of $X_{1}$. Otherwise, when the number of iterations *J* > 1, a sequence of components $g^{\left(1\right)}(n)$ must be obtained to describe a time scale with a clear physical meaning. In this sense, its frequency components are mainly concentrated in the frequency band $\left[f_{\max}-\delta f,f_{\max}+\delta f\right]$, where δf represents the half bandwidth of the main peak in the residual power spectral density. Therefore, a subset $I_{j}(I_{j}=\{i_{1},\cdots ,i_{p}\})$ is created according to all the characteristic groups of the left eigenvector with prominent principal frequencies in the spectrum $\left[f_{\max}-\delta f,f_{\max}+\delta f\right]$ range and one of the characteristic groups with the greatest contribution to the principal peak energy of the selected modal components. Then the corresponding component signals are reconstructed by the diagonal averaging method of the matrix $X_{Ij}=X_{i1}+\cdots +X_{ip}$.

Setting the stop condition of an iteration. The iteratively estimated component sequence ${\tilde{g}}^{\left(j\right)}(n)$ is separated from the original signal and a residual term $v^{\left(j+1\right)}(n)=v^{\left(j\right)}(n)-{\tilde{g}}^{\left(j\right)}(n)$ is obtained. The normalized mean square deviation between the residual term and the original signal is calculated; that is, the normalized mean square deviation between the residual term and the original signal:

(12) $$NMSE^{\left(j\right)}=\frac{\sum_{i=1}^{N}{\left(v^{\left(j+1\right)}(i)\right)}^{2}}{\sum_{i=1}^{N}{\left(x(i)\right)}^{2}}.$$

When the normalized mean square deviation is less than the given threshold *th* = 1%, the whole decomposition process terminates. Otherwise, the residual term is used as the original signal to repeat the above iteration process until the iteration stopping condition is satisfied, and the final decomposition result is obtained:

(13) $$x(n)=\sum_{k=1}^{m}{\tilde{g}}^{\left(k\right)}(n)+v^{\left(m+1\right)}(n)$$

where m is the number of component sequences obtained. It is noteworthy that after each iteration, the energy of the residual $v^{\left(j+1\right)}(n)$ decreases.

**Figure 6:**
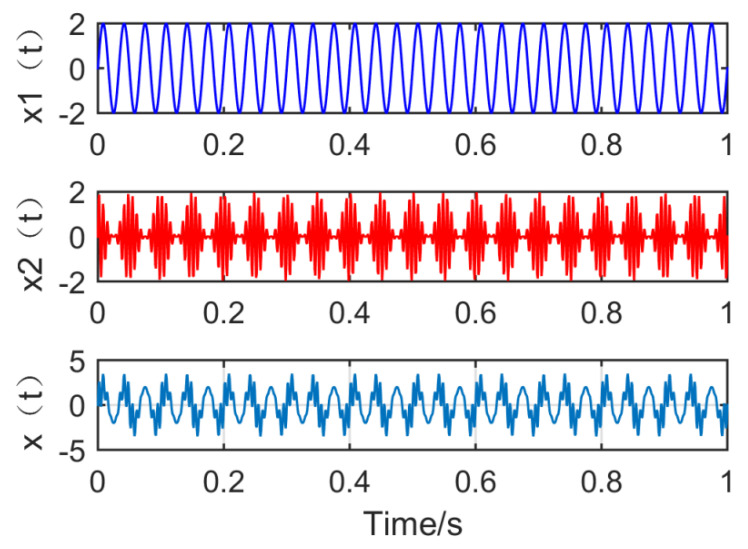
Time domain waveform of the simulation signal.

The SSD method has a higher decomposition accuracy and can better suppress the generation of modal aliasing and pseudo components. In order to compare the decomposition performance of SSD and EEMD, we construct a simulation signal, such as Equation (14), for comparison:

(14) $$\left\{ \begin{array}{l} x_{1}\left(t\right)=2\sin\left(2\pi f_{1}t\right)\\ x_{2}\left(t\right)=\left(1+\cos\left(2\pi f_{n1}t\right)\right)\sin\left(2\pi f_{z}t\right)\\ x\left(t\right)=x_{1}\left(t\right)+x_{2}\left(t\right) \end{array}\right.$$

where $f_{1}=30Hz,f_{n1}=20Hz,f_{z}=150Hz$. The simulation signal consists of a sinusoidal signal and a modulation signal with a modulation source. The waveform of the simulation signal is shown in Figure 6.

**Figure 7:**
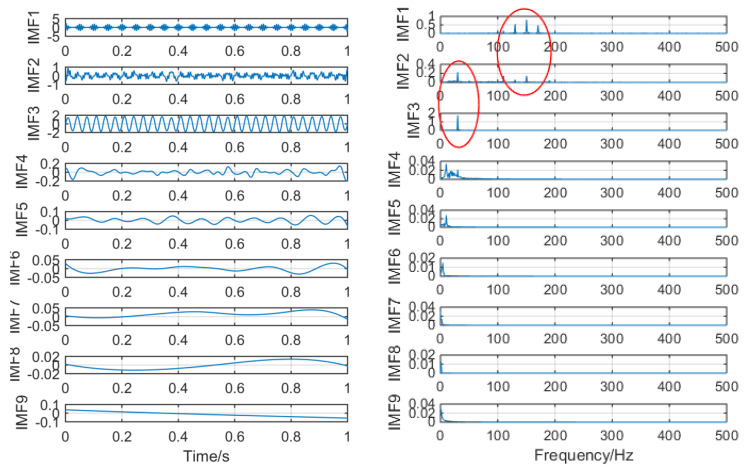
The spectrum of intrinsic mode functions (IMFs) after ensemble empirical mode decomposition (EEMD) and its corresponding spectrum.

**Figure 8:**
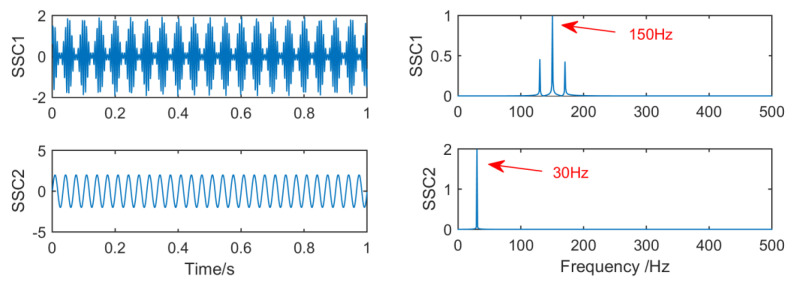
The spectrum of IMFs after singular spectral decomposition (SSD) and its corresponding spectrum.

Figure 7 shows the time–frequency graph of each component of the simulation signal decomposed by EEMD, and Figure 8 shows the time–frequency graph of each component of the simulation signal decomposed by SSD. It can be seen intuitively that the decomposition performance of SSD is more excellent. The decomposed components are almost identical with the simulation signals, and there is no modal aliasing and no false components. However, mode aliasing occurs in IMF, IMF2, IMF3 and IMF4 after EEMD decomposition, which shows that the decomposition performance of SSD is more reliable. Therefore, this paper chooses the SSD method to stratify the original vibration signal.

### 2.3. Principles of DFA

In 1994, Peng et al. [41] proposed the DFA algorithm, which first removed the local trend and then estimated the Hurst index of the object. Initially, this method was used to analyze fractal characteristics of DNA, such as self-similarity, scale invariance and long correlation. With the continuous in-depth study, this method has been widely used in time series data analysis in different fields, such as fault diagnosis of gears and bearings through vibration signals and seismic signal analysis. The research proves that the DFA algorithm is a robust and reliable tool for analyzing non-stationary signals and time series, so this paper chooses the DFA algorithm to divide multiple signal components into noisy signal components and residual signal components. The specific process is as follows:

For the one-dimensional time series X(t), t=1,2,3,⋯,N. The calculation steps of detrended fluctuation analysis are as follows: Calculating the cumulative deviation y(k) of time series x(t), t=1,2,3,⋯,N.

(15) $$y(k)=\sum_{i=1}^{k}\left[x(i)-\bar{x}\right],1\leq k\leq N.$$

The cumulative deviation y(k) is divided into $N_{s}$ non-overlapping windows, where each window contains *s* sampling points, thus $N_{s}=[N/s]$. Each interval can be expressed as a *p*-order trend related to time t, and its corresponding trend equation can be expressed as follows:

(16) $$y_{s}(k)\sum_{j=0}^{p}\beta_{j}t^{j}.$$

In the equation, the coefficient $\beta_{j}(j=0,1,2,\cdots ,p)$ is obtained by the least squares fitting method, where *p* is the fitting order.

Eliminate the trend term $y_{s}(k)$ of y(k) of each window time series:

(17) $$\Delta y_{s}(k)=y(k)-y_{s}(k).$$

The second-order wave function of time series $\Delta y_{s}(k)$ is calculated by using the following equation:

(18) $$F(s)=\sqrt{\frac{1}{N}\sum_{i=1}^{N}{\left(\Delta y_{s}(k)\right)}^{2}}.$$

The window size s increases with a certain step size and repeats the (12)–(14) steps to obtain the curve of function F(s) varying with the window size s. If the curve obeys the power law relation, it exists as

(19) $$F(s)\propto s^{\alpha }\Rightarrow F(s)=AS^{\alpha }.$$

It can be found from the above equation that lg(F(s)) and lgs are linearly correlated, and their scaling index α is the slope that can be obtained by the least square method:

(20) lg(F(s))=lgA+αlgs.

### 2.4. Principles of the Autoregressive (AR) Model

The AR model is widely used in signal denoising. The basic idea of an AR model is to describe the time-varying and interrelated data series by using relevant mathematical models, and to analyze and study them, so as to understand the internal structure and complexity of dynamic data in essence, so as to achieve the best prediction effect of the data. Wang et al. [42] designed a filter based on an AR model to separate vibration impulse signals generated by local cracks in gear teeth, and used the kurtosis of prediction error signals of the AR model as fault characteristic parameters. Compared with the traditional residual vibration signal of gears, which eliminates meshing resonance frequency, an AR model’s predictive error signal can reflect the tooth defect more clearly. In this paper, an autoregressive (AR) model is used to filter the components of noisy signals. The impulse components of vibration signals are separated, and the first noise reduction is completed. The specific process is as follows:

For the zero-mean discrete sequence {x(n)}, $x_{i}$ can be linearly expressed by the first i values of the signal, and the k-th order autoregressive model AR(k) can be obtained by the idea of multiple linear regression:

(21) $$x_{i}=\sum_{j=1}^{k}a_{j}x_{n-j}+e_{n}.$$

where $x_{i}$ is the i-th time series point to be predicted; $a_{j}$ is the j-th coefficient of AR model; k is the order of AR model; and $e_{n}$ is the white noise sequence with 0 mean variance $\sigma^{2}$.

For the solution of the AR model coefficients, the autocorrelation coefficients of signals are often used. The model satisfies the Yule–Waker equation:

(22) $$r_{x}(k)+\sum_{i=1}^{j}a(l)r_{x}(j-l)=\left|b{\left(0\right)}^{2}\right|\delta (j).$$

Its matrix form is

(23) $$\left[\begin{array}{cccc} r_{x}(0) & r_{x}(-1) & \cdots & r_{x}(p-1)\\ r_{x}(1) & r_{x}(0) & \cdots & r_{x}(2-p)\\ \vdots & \vdots & \ddots & \vdots \\ r_{x}(p-1) & r_{x}(p-2) & \cdots & r_{x}(0) \end{array}\right]\left[\begin{array}{c} a(1)\\ a(2)\\ \vdots \\ a(p) \end{array}\right]=\left[\begin{array}{c} r_{x}(1)\\ r_{x}(2)\\ \vdots \\ r_{x}(p) \end{array}\right].$$

In the equation $r_{x}(p)=\frac{1}{n}\sum_{i=0}^{n-1}x(i)x(i-p)$, 0≤p≤k−1, and n is the number of signal points sampled.

The order determination of the AR model is very important, as too large an order will produce pseudo-spectral peaks and unstable statistical values, whereas too small an order will produce the smoothing effect of spectral peaks. Minimum Information Criterion (AIC) is widely used. Its criterion function is

(24) $$AIC(k)=N\ln(\sigma^{2})+2k.$$

The value of AIC(k) decreases with the increase of order k from 1. When the order k reaches a certain order $k_{k}$, the value of AIC(k) tends to be stable and $k_{k}$ is the best order of AR model.

### 2.5. Principles of New Methods

The purpose of this paper is to optimize the Med algorithm and improve the noise reduction efficiency of MED. At present, Med has two main defects; that is, poor anti-noise ability and the filter size need to be determined manually. Firstly, aiming at the defect of the bad anti-noise ability of MED, this paper proposes a preprocessing method for the original signal. The SSD algorithm has a higher decomposition accuracy than EEMD, so that the SSD algorithm is used to layer the original signal. However, it is easy to produce high-frequency pseudo components when using the SSD algorithm to process signals, and it is greatly interfered in the strong noise environment, so this paper chooses to use a Gaussian white noise assisted SSD algorithm to make up for the shortcomings of SSD. After the original signal is layered successfully, the DFA algorithm is used to distinguish the layered signal components; that is, the original signal is divided into noisy signal components with a lot of noise and residual components with less noise. After that, the noisy component is de-noising, while the residual component is less noisy. In order to ensure the integrity of the fault features, the residual component is not processed. Secondly, in order to solve the problem that the size of the MED filter is not self-adaptive, this paper proposes a method to select the optimal filter size through the optimization algorithm of firefly. Finally, the noisy and residual components are reconstructed to get the final result.

Step 1: Signal Preprocessing

The original signal is decomposed into a series of signal components by noise-assisted SSD. In SSD, modal aliasing and high-frequency pseudo components occur because of its vulnerability to noise. For this reason, adding white noise into the original signal not only makes use of the uniform distribution of the white noise spectrum to automatically distribute signals of different time scales to the appropriate reference scale, but also makes use of the zero-mean characteristic of white noise. After many times of average, the noise cancels each other, thus eliminating the influence of noise on signal components. Through this white noise processing, the problem that the SSD method is prone to generate high-frequency pseudo-components has been greatly improved. The steps are as follows: Given an original signal x(t), white noise $n_{j}(t)$ with a zero mean and constant standard deviation of amplitude is added, in which j=1,2,3⋯M, *M* being the number of iterations. 

So, the new signal is

(25) $$x_{j}(t)=x(t)+a_{0}n_{j}(t)$$

where $a_{0}=\varepsilon_{0}std(s)/std(n_{j}(t))$, $\varepsilon_{0}$ is the standard deviation and s is the original signal.

The SSD algorithm is used to decompose the signal several times, and the result shows the first signal component decomposed in the *j*-th experiment. If *j* is less than *M*, repeat the previous step.

According to the principle that the statistical mean of the uncorrelated random sequence is zero, the above-mentioned signal components are averaged in general, which can eliminate the influence of multiple white noises on the signal components. Finally, the obtained signal component is

(26) $$c_{i}=\left(\sum_{j=1}^{M}c_{i,j}\right)/M$$

where the output $c_{i}(i=1,2,3\cdots I)$ is the *i*-th signal component obtained.

DFA is used to screen and process the signal components, and the Hurst index H(*q*) of each signal component is obtained. If 0 < H(*q*) < 0.5, the signal components have an inverse correlation; that is, the smaller the value of the friction vibration is, the stronger the inverse correlation. When H(*q*) = 0.5, the time series is irrelevant, and the friction vibration has no obvious forward and backward trend. When 0.5 < H(*q*) < 1, the time series has a correlation; that is, the larger the value, the stronger the continuity. The threshold is usually determined by the Hurst exponent of white noise α=0.5.

**Figure 9:**
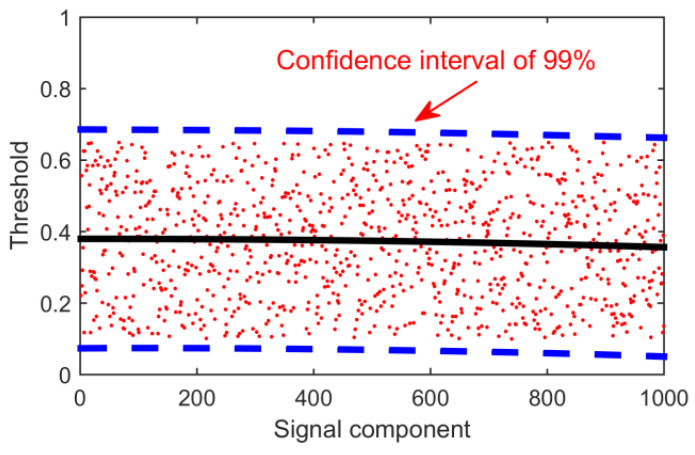
Hurst index distribution map.

However, in many practical operations, it is found that the decomposition algorithm produces modal aliasing. In other words, the overlapping of two signal components results in multiple fault features in one signal component. So sometimes the Hurst index with a noise component is slightly larger than 0.5, ranging from 0.5 to 0.7. In order to deal with this situation, 1000 experiments were carried out in this paper. By choosing completely random signals and adding random noise, the Hurst index of the decomposed noisy signal components was calculated. Finally, the coordinate axis is established, the 1000 points are fitted by a curve, and the upper boundary of 99% confidence interval is selected as the final threshold. As shown in the following Figure 9, the threshold should be 0.7. 

The specific screening steps are as follows:

The signal component whose scale index is lower than the threshold value is the noisy component; and the signal component, whose index is higher than the threshold, is the residual component.

Finally, all the signal components are divided into two parts, as follows:

(27) $$x(t)=\sum_{i=1}^{m}SSC_{i}(t)+\sum_{i=m+1}^{q}SSC_{i}(t).$$

**Figure 10:**
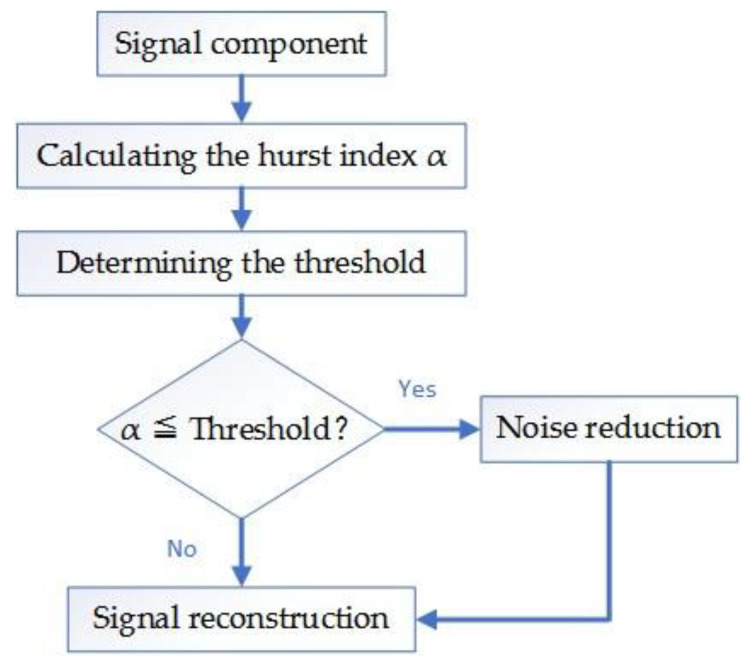
Flow chart of the screening signal component.

The flow chart of the screening signal component is as follows (Figure 10):

Step 2: Adaptive MED algorithm

After processing the signal component by using the autoregressive (AR) model, the adaptive MED algorithm proposed in this paper is used to process the signal component for the second time, and then reconstruct it with the original residual signal to get the final result. The specific methods are as follows:

In this paper, envelope spectral entropy is chosen as the fitness function of the FA algorithm to optimize filter length parameters. The theory of envelope spectral entropy is as follows:

When there are local damages or defects in rolling bearings, impulsive force will be generated during the load-bearing operation, which will stimulate the high-frequency natural vibration of bearings. The inherent vibration can be regarded as the high frequency carrier of the bearing vibration signal, while the periodic shock is the low frequency modulation signal. The final vibration waveform of the bearing is a complex amplitude modulation wave. The envelope demodulation analysis based on Hilbert transform is an effective method to analyze the amplitude modulation signal. 

The Hilbert transform h(t)=H(x(t)) of signal x(t) is defined as

(28) $$h(t)=\frac{1}{\pi }\int_{-\infty }^{+\infty }\frac{x(\tau )}{t-\tau }d\tau .$$

x(t) and h(t) can form new composite signals:

(29) z(t)=x(t)+jh(t).

Envelope signal is defined as

(30) $$E(t)=\left|z(t)\right|=\sqrt{x^{2}(t)+h^{2}(t)}.$$

Through envelope demodulation analysis, the low frequency modulation signal in the modulation signal, namely envelope signal, can be obtained. Envelope spectrum analysis can effectively extract the fault frequency components of rolling bearing vibration signals, which is the most widely used analysis method in rolling bearing vibration analysis. The degradation of rolling bearing performance will inevitably bring about changes in the internal characteristics of vibration signals. In order to effectively measure this change, the envelope spectrum analysis and information entropy are combined; that is, envelope spectrum entropy:

(31) $$\{\begin{array}{c} H_{e}=-\sum_{i=1}^{N}p_{i}*\ln p_{i}\\ p_{i}=HX(i)/\sum_{j=1}^{N}HX(j)\\ \sum_{i=1}^{N}p_{i}=1 \end{array}$$

where HX(i) is the envelope spectrum of vibration signal $\left\{x_{i}\right\},\;i=1,2\cdots ,N$, and $H_{e}$ is the envelope spectrum entropy.

To avoid the influence of data length on the results, the normalized envelope spectral entropy is defined:

(32) $$H_{e}=-\frac{1}{\ln N}\sum_{i=1}^{N}p_{i}*\ln p_{i}$$

It can be seen that envelope entropy measures the uniformity of frequency distribution of the envelope signal and reflects the complexity of the signal in the envelope domain. Envelope spectrum entropy depends only on the frequency distribution of the envelope signal but has nothing to do with the strength of the signal. Therefore, in order to make the denoised signal highlight a more effective continuity period, for envelope spectrum entropy, the smaller the better.

Let $F(x)=\min H_{e}(x)$, the key of MED denoising is how to select the filter size L. If the filter size is different, the denoising results will be different. At the same time, it should be noted that the larger the L value selected, the longer the computation time needed for noise reduction. In this paper, the envelope spectrum entropy is used to measure the effect of signal de-noising, that is to say, the signal is de-noised by MED with different filter sizes, then the envelope signal is obtained by Hilbert transform, and then the envelope spectrum and envelope spectrum entropy are calculated.

FA optimization algorithm uses firefly individuals to simulate points in search space, and uses the phototaxis of firefly itself to transform the optimization problem into finding the brightest firefly in the firefly population. Each iteration finds the brightest firefly and updates the position of firefly through attraction and movement between fireflies.

The rule that firefly i moves and updates to brighter firefly j is

(33) $$X_{i}=X_{i}+\beta (r)\times (X_{j}-X_{i})+\alpha \times (rand()-0.5),$$

where $X_{i}$ and $X_{j}$ are the spatial locations of firefly i and j, respectively; $\beta (r)=\beta_{0}e^{-\gamma r_{ij}^{2}}$ is the attraction of firefly j to firefly i; $r_{ij}=\left\|X_{i}-X_{j}\right\|$ is the distance between firefly i and j; $b_{0}$ is the attraction of $r_{ij}=0$; g is the absorption coefficient of light intensity; α is the step factor, α∈[0,1]; and (rand()−0.5) is the interference term to avoid the FA algorithm falling into local optimum.

**Figure 11:**
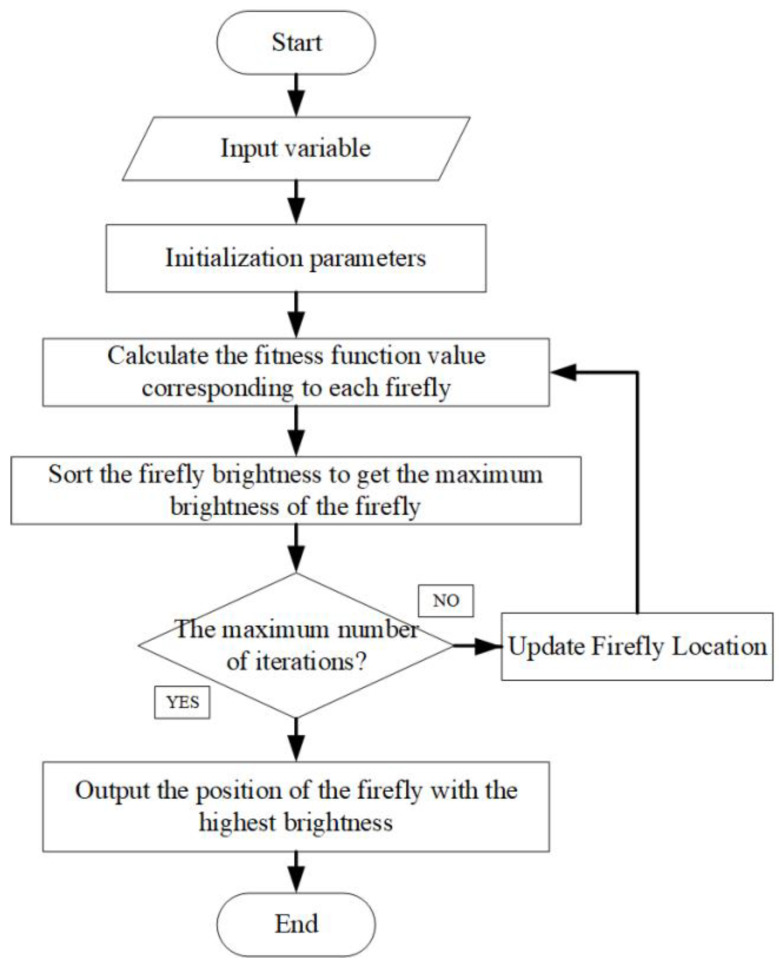
Flow chart of the firefly optimization algorithm.

**Figure 12:**
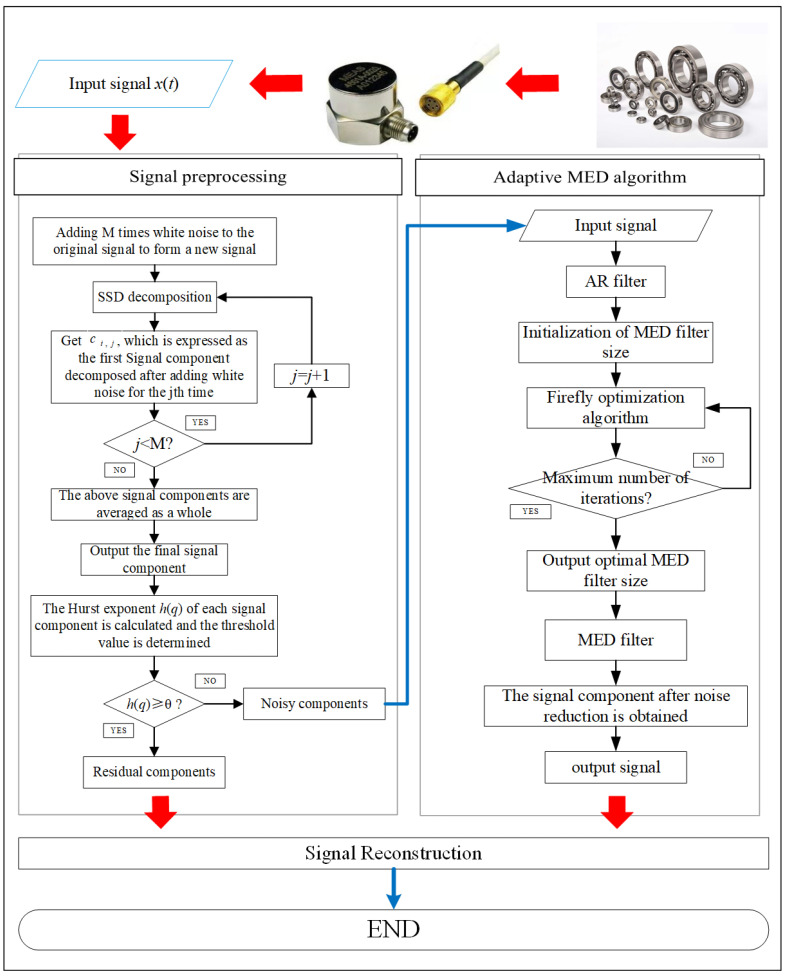
The novel method flow chart.

The equation $F(x)=\min H_{e}(x)$ is taken as the objective function of the firefly algorithm to optimize the filter size. The optimal result of the firefly algorithm is the position $X_{best}(X_{i}^{*})$ of the firefly with the greatest brightness. Among them, $X_{i}^{*}$ is the required filter size. The steps of filter size optimization based on the firefly algorithm are as follows:

(1) Initialization FA parameters: Number of fireflies N, initial attractiveness $\beta_{0}$, step size factor α, initial position of fireflies $X_{i}$ and maximum iteration times T.
(2) The brightness of each firefly is calculated and sorted: The fitness $F_{i}$ corresponding to each firefly is calculated, and fitness $F_{i}$ is taken as the brightness of the corresponding firefly and sorted to get the position of the firefly with the greatest brightness.
(3) Judging whether the iteration is over: If the algorithm reaches the maximum iteration number T, then the algorithm goes to (4), otherwise it goes to (5).
(4) The position and brightness of the firefly with the greatest brightness are output, and the obtained $X_{i}^{*}$ is used as the optimal scale of the filter.
(5) Update the location of fireflies: Update the location of fireflies according to Equation (32). The flow chart of MED filter size optimization algorithm based on the firefly algorithm is shown in Figure 11, and the flow chart of the new method is shown in Figure 12.

The adaptive MED algorithm is used to denoise the noisy signal components and reconstruct the signal with the residual components:

(34) $$\hat{x}(t)=\sum_{l=m+1}^{q}\left(MED\;(SSC_{l}(t))+\sum_{l=q+1}^{n}SSC_{l}(t)\right).$$

The signal components *m* + 1 to q are the noisy signal components, and the signal components *q* + 1 to *n* are the residual components, $\hat{x}$ being the final result.

## 3. Simulated Signal Analysis of a Gearbox Compound Fault

### 3.1. Construction of Simulated Signals 

In order to verify the effectiveness and superiority of the proposed method, the following signals are constructed and simulated. Vibration signals of bearing faults are usually expressed as periodic shocks, as shown in Equation (35):

(35) $$\begin{array}{l} x(t)=x_{1}(t)+nosie\\ \{\begin{array}{c} nosie=0.5\times randn(t)\\ x_{1}(t)=A_{m}\times \exp(-g/T_{m1})\sin(2\pi f_{1}t) \end{array} \end{array}$$

where $A_{m}$ is the magnitude of impact, g is the damping coefficient, $T_{m1}$ is the period of impact and $f_{1}$ is the frequency conversion of axis. Among them, $f_{1}$ is 150Hz. Other parameters are shown in Table 1.

**Figure 13:**
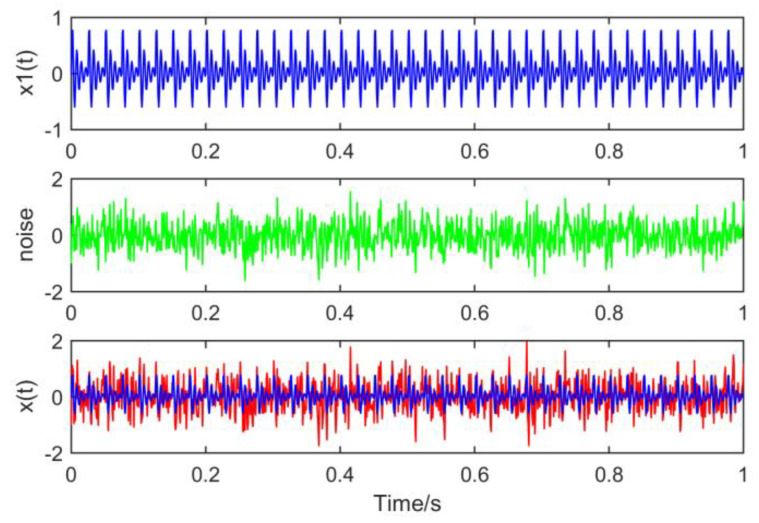
Time domain waveforms of the composite signal.

Set the sampling point *N* to 1000 and the sampling frequency *Fs* to 1000 Hz. The waveforms of the constituent signals $x_{1}(t)$, noise and time domain simulation signals x(t) are drawn respectively, as shown in Figure 13:

### 3.2. Comparison of Decomposition Results of Different Algorithms

**Figure 14:**
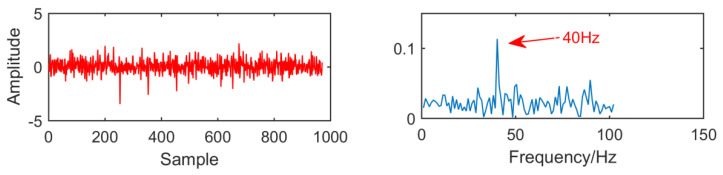
Time domain waveform and envelope diagram obtained by minimum entropy deconvolution (MED).

In order to compare with the new method, we first use the MED algorithm to process the original signal. The results are shown in Figure 14. It can be seen that MED is greatly disturbed by noise in strong noise environment. Although the fault characteristic frequency 40 HZ has been successfully extracted, but the envelope diagram has no multiple frequency of the fault characteristic frequency. The effect is not very satisfactory.

**Figure 15:**
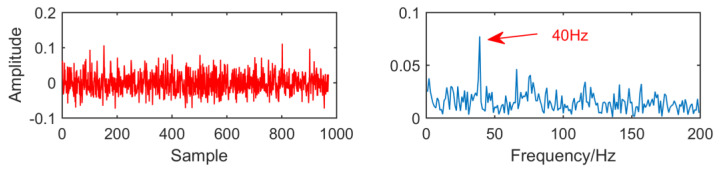
Time domain waveform and envelope diagram obtained by multipoint optimal minimum entropy deconvolution adjusted (MOMEDA).

Figure 15 is the time domain and envelope diagram of the processing results of the above simulation signals by traditional multipoint optimal minimum entropy deconvolution adjusted (MOMEDA). From this figure, we can see that although the modulation frequencies of 40Hz is successfully extracted, they are greatly affected by noise and the results are not ideal.

**Figure 16:**
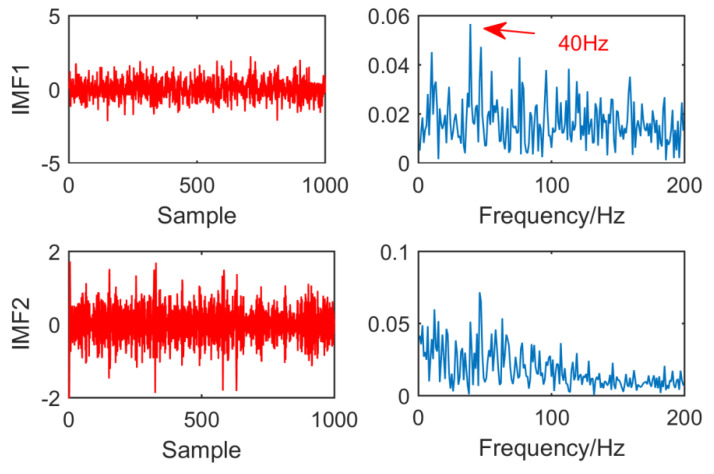
Time domain waveform and envelope diagram obtained by EEMD.

Figure 16 is the result of EEMD processing of the original signal. Because the low frequency component of EEMD algorithm does not contain fault features. In order to facilitate the observation of fault features, the first two layers of IMF are selected for observation. It can be observed that in IMF1 and IMF2, EEMD can extract fault features of 40 Hz, but the noise is large, and the effect is not ideal.

**Figure 17:**
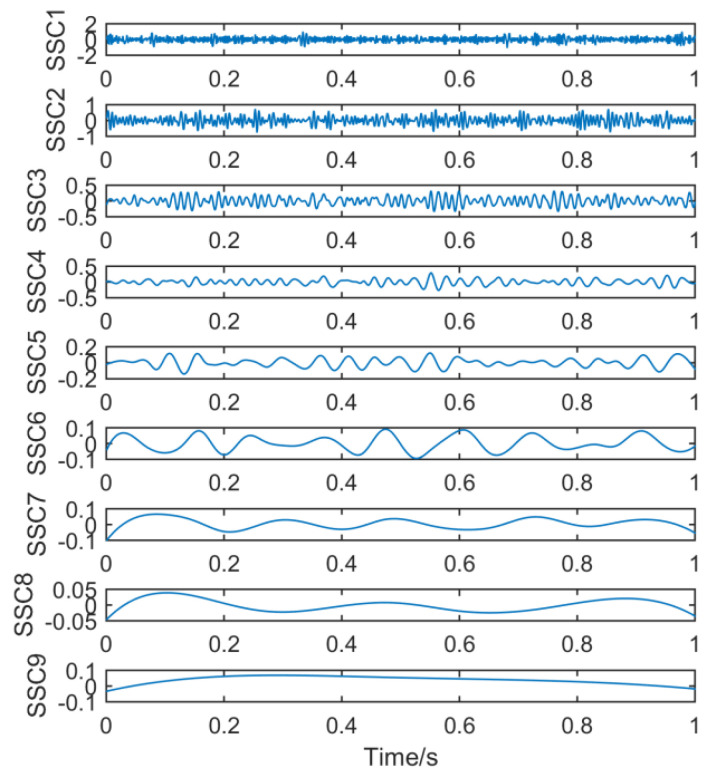
Time domain waveform obtained by noise-assisted SSD.

Finally, the new method is used to process the signal. Figure 17 shows nine sets of signal components after the original signal is processed by the noise-assisted SSD algorithm.

**Figure 18:**
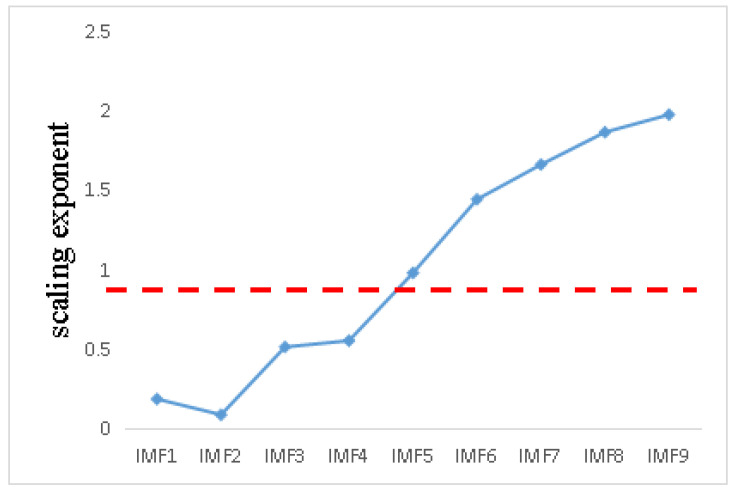
Scaling index distribution line chart.

The Hurst index of nine signal components is calculated. As shown in Figure 18, the threshold value is still 0.7, and the signal component whose scaling index is less than 0.7 is the signal component with noise. It needs to be de-noised by adaptive MED algorithm. The residual component is the low-frequency component and the signal index is higher than the threshold value.

**Figure 19:**
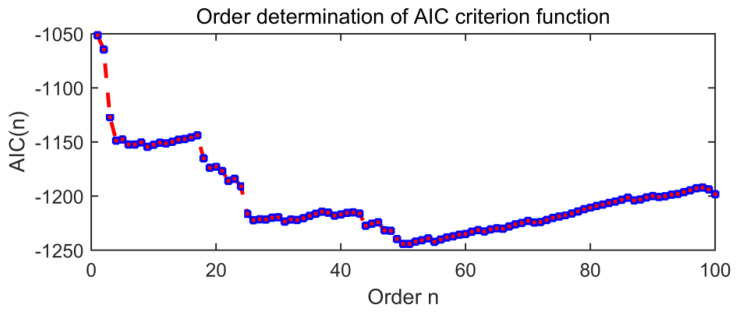
The order determination diagram of the AIC criterion function.

**Figure 20:**
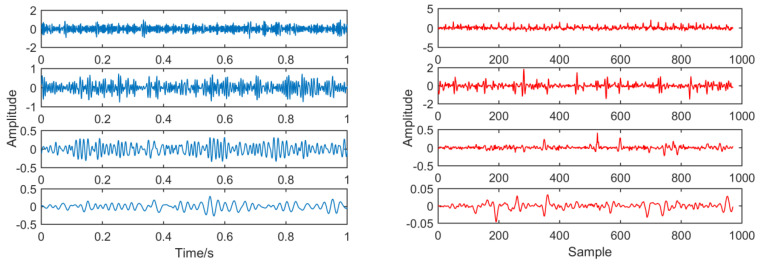
Contrast before and after noise reduction.

Figure 19 is the order determination diagram of the AIC criterion function, and the order of the AR model is 50. Figure 20 is a time domain image of noise signal components through AR and adaptive MED filters, in which the size of the MED filter is adaptively selected to 32.

**Figure 21:**
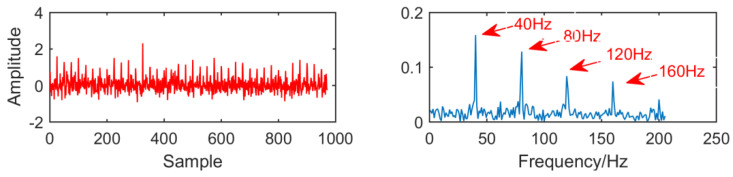
Time domain waveform and envelope diagram obtained by the new method.

For our new decomposition method, we can see from Figure 21 that the envelope diagram is successfully extracts the modulation frequency of 40 Hz, as well as its double and triple frequencies, and the effect is clearly visible. It can be seen that in a strong noise environment, compared with MED and MOMEDA, the anti-noise ability of this new method has been greatly improved, and noise reduction efficiency has been significantly improved.

**Figure 22:**
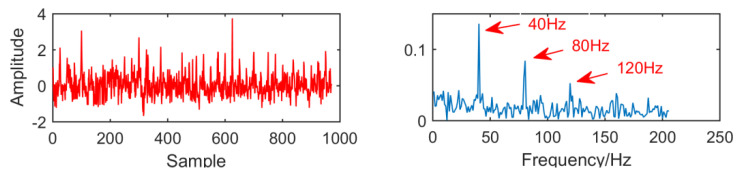
The results when the amplitude of noise is 0.7.

In order to observe the signal processing results of the new method under different SNR, we adjust the noise amplitude to 0.7, and the final result is shown in Figure 22. It can be seen that the fault signal frequency 40 Hz and its multiple frequency are successfully extracted in the envelope diagram.

## 4. Experimental Verification

**Figure 23:**
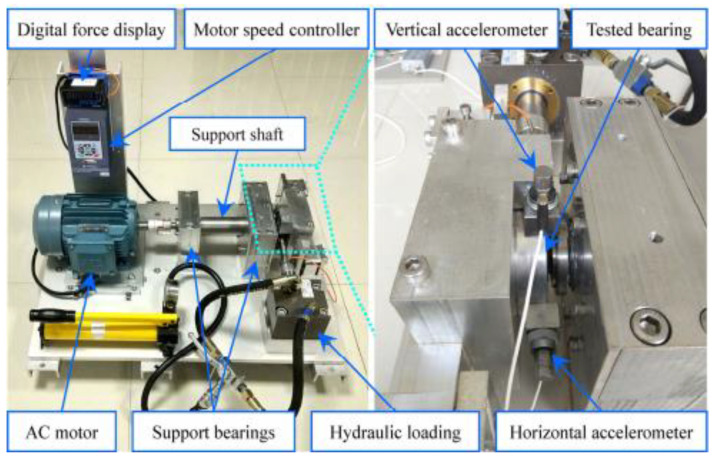
Rolling bearing test bench.

**Figure 24:**
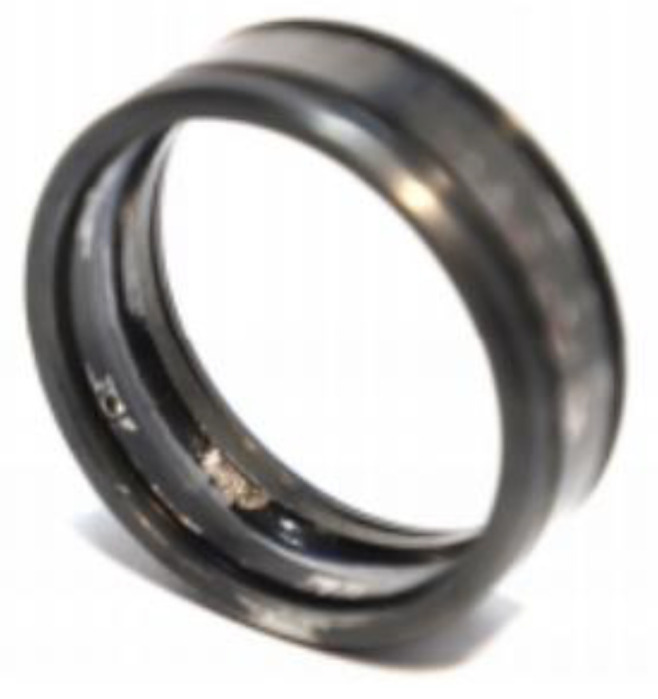
Photo of failed bearing (outer ring worn).

In order to verify the feasibility and superiority of the new method in engineering application, the data provided by XJTU and Changxing Suyang Science and Technology Co., Ltd, are selected for analysis. The test bearing model is LDK UER204. Its parameters are bearing pitch diameter D = 34.55 mm, rolling body diameter d = 7.92 mm, number of rolling bodies n = 8, rotating speed n = 2400, sampling point 4096, sampling frequency 25,600 Hz, and calculating inner ring fault characteristic frequency f = 123.2 Hz. As shown in Figure 23, the bearing test bench is composed of a supporting shaft, motor speed controller, AC induction motor, hydraulic loading system, etc. Figure 24 is the photo of the failed bearing. Various parameters are shown in Table 2.

**Figure 25:**
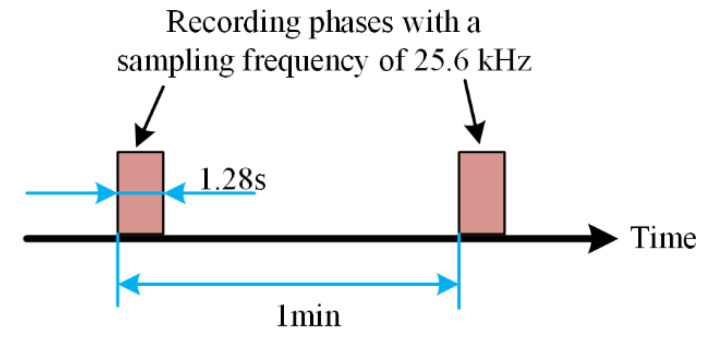
Vibration signal sampling setting.

In order to collect the vibration signal of the tested bearing, as shown in Figure 25, place two PCB 352C33 accelerometers in the 90° position of the tested bearing housing; that is, one is installed on the horizontal axis and the other is installed on the vertical axis. The sampling frequency is set to 25.6 kHz. 32,768 data points (i.e., 1.28 s) are recorded for each sampling, with a sampling period of 1 min.

**Figure 26:**
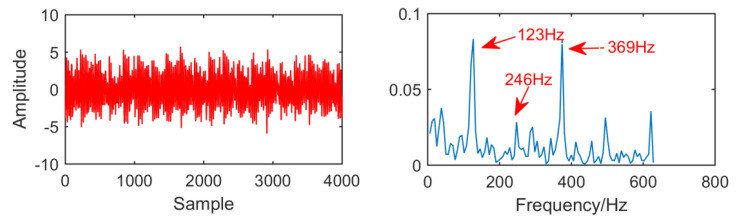
Time domain waveform and envelope diagram waveform of the fault signal.

The fault signal of the outer ring of rolling bearing is shown in Figure 26. It can be seen that the amplitude of the noise is larger than that of the outer fault signal, and the outer fault signal is submerged by the noise. Through envelope spectrum analysis of the time domain signal, it can be observed that although the frequency doubling is 123 Hz, the extraction of the frequency doubling is not very ideal and cannot accurately describe the fault frequency.

**Figure 27:**
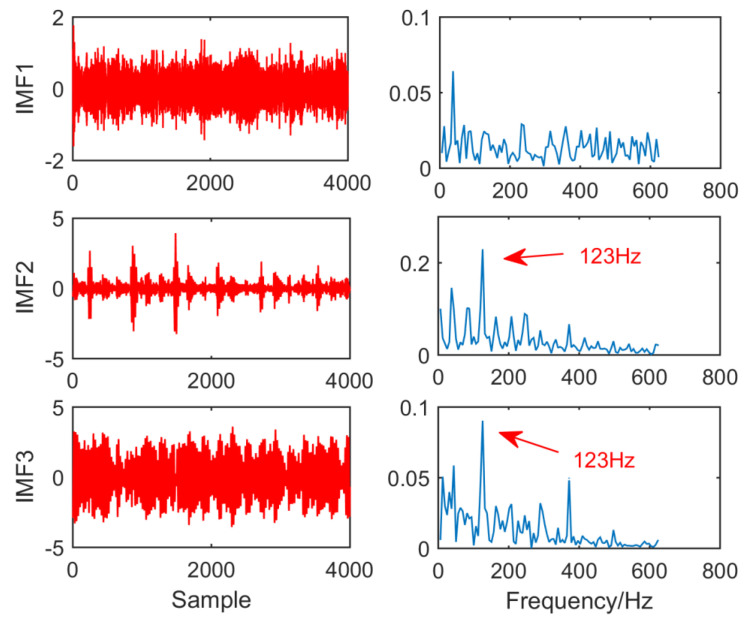
Time domain waveform and envelope diagram waveform obtained by EEMD.

Figure 27 is the time domain diagram and envelope spectrum of EEMD after fault vibration signal processing. It can be observed that no fault feature can be extracted in imf1, and in IMF2 and IMF3, EEMD can extract fault features of 40 Hz, but only extracted one frequency of the fault feature. The result does not accurately describe the fault feature.

**Figure 28:**
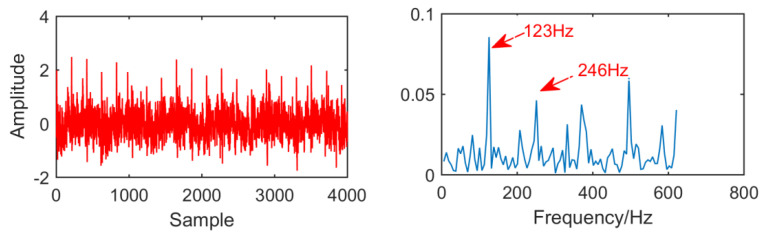
Time domain waveform and envelope diagram waveform obtained by MED.

Figure 28 is the time domain diagram and envelope spectrum of MED after fault vibration signal processing. Compared with Figure 22, the noise amplitude of the fault vibration signal after MED treatment is much lower and the pulse signal is highlighted, but the envelope spectrogram only extracts the double and double frequencies of the fault features, and it is not very clear. Therefore, it can be concluded that the noise reduction effect of MED is not very ideal.

**Figure 29:**
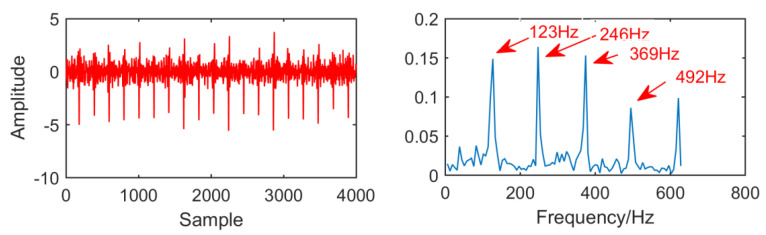
Time domain waveform and envelope diagram waveform obtained by the new method.

In Figure 29, as for the result of the new method, adaptive selection of filter size to 60 and the order of AR model is 99. In the envelope spectrum, the fault frequencies and multiple frequency 123 Hz, 246 Hz, 369 Hz and 492 Hz are successfully extracted, and the fault frequencies are relatively clear. Generally speaking, this method improves the MED algorithm. In order to observe the improvement effect of the new method more conveniently, the envelope spectral entropy corresponding to each method is listed as shown in Table 3.

## 5. Conclusions

In order to improve the efficiency of MED, a novel method based on MED for faults of the gearbox is proposed to overcome the shortcomings of poor anti-noise ability and non-adaptive filter length in fault diagnosis. It provides a new idea for an adaptive signal processing method.

The simulation signal of rolling bearing fault and the measured signal of engineering are used to analyze the new method. At the same time, by comparing with MED and other traditional methods, the reliability and validity of this method in rolling bearing fault diagnosis are verified. The final results show that the new method can extract fault features more effectively than MED and reduce the interference of noise on fault diagnosis. However, the proposed method still has some shortcomings. For example, there are still some parameters in the new method to select empirical values, which will be continuously improved in the future research. In future research, we will further study the adaptive selection of parameters and keep learning the latest fault diagnosis methods.

## Figures and Tables

**Table 1:** The parameters of the simulation signal.

Parameter	$T_{m1}$	$A_{m}$	g	AR Order	Iteration Times M
Numerical values	1/40	1	0.1	50	50

**Table 2:** Parameters of the tested bearings.

**Parameter**	**Outer Race Diameter**	**Bearing Mean Diameter**	**Number of Balls**	**Load Rating (Static)**
Numerical values	39.80 mm	34.55 mm	8	6.65 KN
**Parameter**	**Inner Race Diameter**	**Ball Diameter**	**Contact Angle**	**Load Rating (Dynamic)**
Numerical values	29.30 mm	7.92 mm	0°	12.82 KN

**Table 3:** Envelope spectrum entropy corresponding to each method.

	Original Signal	MED	New Method
Envelope spectral entropy	7.1557	6.9725	6.6656
